# Does intestinal anastomosis in resection of colon cancer have a significant impact onto early postoperative outcome and long-term survival?

**DOI:** 10.1515/iss-2023-0026

**Published:** 2023-11-03

**Authors:** Ellen Hajduk, Frank Meyer, Ronny Otto, Roland Croner, Karsten Ridwelski

**Affiliations:** Department of General and Abdominal Surgery, Magdeburg Municipal Hospital (Klinikum Magdeburg GmbH), 39130 Magdeburg, Germany; Institute of Quality Assurance in Operative Medicine, Otto von Guericke University, Magdeburg, Germany; Department of General, Abdominal, Vascular and Transplant Surgery, Otto-von-Guericke University with University Hospital, 39120 Magdeburg, Germany

**Keywords:** colon cancer, intestinal anastomosis, urgency of surgical intervention, early postoperative outcome, long-term oncosurgical outcome, survival (rate)

## Abstract

**Objectives:**

To investigate the influence of anastomosis on the early postoperative and long-term oncological outcomes of patients with primary colon carcinoma (CA).

**Methods:**

All consecutive patients with the histologically diagnosed primary colon CA (design, prospective multicenter observational study) were registered with regard to patient-, diagnostic-, tumor (Tu) finding-, and treatment-related aspects using a computer-based registry with 60 items to characterize early postoperative and long-term oncological outcome.

**Results:**

*Basic data*: From 2010 to 2016, data from a total of 14,466 patients were documented (mean age, 72.8 [range, 22–96] years; sex ratio, m:f=7,696:6,770). – 717/14,466 patients (4.96 %) were included in a matched-pair analysis. The majority of these patients underwent elective surgery (*n*=12,620 patients; 87.2 %) regardless of whether a bowel anastomosis or an ostomy was created. In emergency surgery, a bowel anastomosis was possible in a large proportion (*n*=1,332 patients [72.1 %]). In contrast, in 514 patients (27.9 %) who underwent emergency surgery, an ostomy was created. Interestingly, ostomy had to be created even less frequently in patients who had undergone planned surgery (*n*=366 [2.5 %]). – *Early postoperative outcome*: Cases of postoperative mortality were mainly due to general complications. Especially among the patients treated in an emergency situation without intestinal anastomosis, a high proportion died of their pre-existing condition (17.0 %). Patients who underwent ostomy creation or emergency surgery had a worse risk profile (incl. arterial hypertension, diabetes mellitus, and secondary cardiac or renal diseases) which led to the decision to operate without anastomosis. Furthermore, data show no matter which technique had been used, patients that had undergone surgical intervention without anastomosis were more likely to develop complications. – *Long-term oncosurgical outcome*: The most important factors influencing long-term survival were age, resection status, and tumor stage (according to TNM and UICC). The more advanced the tumor growth, the lower the long-term survival. Patients categorized with the same tumor stage, age, and risk factors had a better chance of survival, if they underwent elective surgical intervention and with intestinal anastomosis. Interestingly, the multivariable analysis showed that older patients and such with distant metastasis benefit from a discontinuity resection.

**Conclusions:**

The association of intraoperative and postoperative complications with increased postoperative mortality, as well as preexisting risk factors and perioperative complications is in line with findings of current studies. Furthermore, current studies also agree that older patients and such with reduced general condition benefit from discontinuity resection.

## Introduction

According to the guidelines, the treatment of colon carcinoma (colon CA) primarily consists of surgical tumor resection with associated lymphatic drainage area and tumor-free resection margins (R0 resection) [1], with sufficient safety margins. In this context, surgery with primary anastomosis is distinguished from discontinuity resection [2]. If the (local) tumor-associated resectability and functional operability (operation duration, procedural invasiveness, etc.) permits, a continuity resection should be performed. If this is not the case, a discontinuity resection and creation of an artificial intestinal outlet should be performed [3], in particular, in case of obstruction [4], as bridge to surgery [5, 6] or in patients, who have an increased risk of non-reversal of the ostomy [7]. In contrast, *no ostomy* is considered one of the 6 health care parameters (in addition, surgery within 6 weeks, radical resection, lymph node (LN) yield ≥12, no adverse outcome and colonoscopy before/after surgery within 6 months), which belong to the definition of “textbook outcome” [8].

The present prospective observational study aimed to analyze the influence of surgical technique, in particular, the possible creation of intestinal anastomosis (primary endpoint), and surgical urgency (elective vs. emergency – as secondary endpoint) onto the early postoperative and long-term oncological outcomes of patients with primary colon CA on the basis of a representative number of patients over a defined period of time.

## Methods

Colon CA study (histologically confirmed primary colon CA) at the Institute of Quality Assurance in Operative Medicine – Otto-von-Guericke University of Magdeburg (Germany).

Over a 7-years study period, all consecutive patients with histologically confirmed primary colon CA were registered in this multicenter clinical systematic prospective multicenter observational study (study design) as a contribution of research on clinical care to surgical quality assurance in everyday clinical practice and documented in a computerized data registry file. The method has been adequately reported elsewhere [4, 9]. Patients were included in a matched-pairs analysis with respect to pT, pN, R, and M status, Union for International Cancer Control (UICC) stage, grade, risk factors, age, sex, body mass index (BMI), and location of colon CA as follows:

(i) with vs. without intestinal anastomosis – primary study endpoint (as well as)
(ii) elective vs. emergency surgery – secondary study endpoint

and patients were operated on (formation of a “statistical twin group”).

Early postoperative outcomes are described as postoperative morbidity (further characterized by overall and specific [surgery-associated] complication rates) and 30-d or in-hospital mortality. Long-term oncological outcome is defined as 5-year (yr) overall survival, 5-yr tumor-free survival, and 5-yr local recurrence rate.

### Statistics

Data management and statistical analyses were performed with the statistical program SPSS (version 21; SPSS Systems; Chicago/IL, USA). Continuous variables such as times and magnitudes are described by the usual measures of mean and standard deviation as well as minimum, lower quartile, median, upper quartile, and maximum. Categorical variables are represented by their absolute and relative frequencies. In addition, the data were examined by survival analysis means using the Kaplan–Meier test, and the respective event probabilities are represented by a Kaplan–Meier curve.

Intergroup differences in survival were compared by the log-rank test. Furthermore, the median survival time and corresponding 95 % confidence interval were calculated. Cox regression was used to analyze the survival data. To reject the null hypothesis, a p-value <0.05 was considered statistically significant.

Furthermore, a matched-pair analysis was applied to ensure comparability between the two groups. In particular, a prospensity-score matched-pair analysis was used to select patients from one group (patients with anastomosis) with regard to certain characteristics and the same patients from another group (patients without anastomosis). The characteristics were sex, age, risk factors, tumor node metastasis stage, and urgency (elective vs. emergency).

## Results

### Basic data

**Figure 1:**
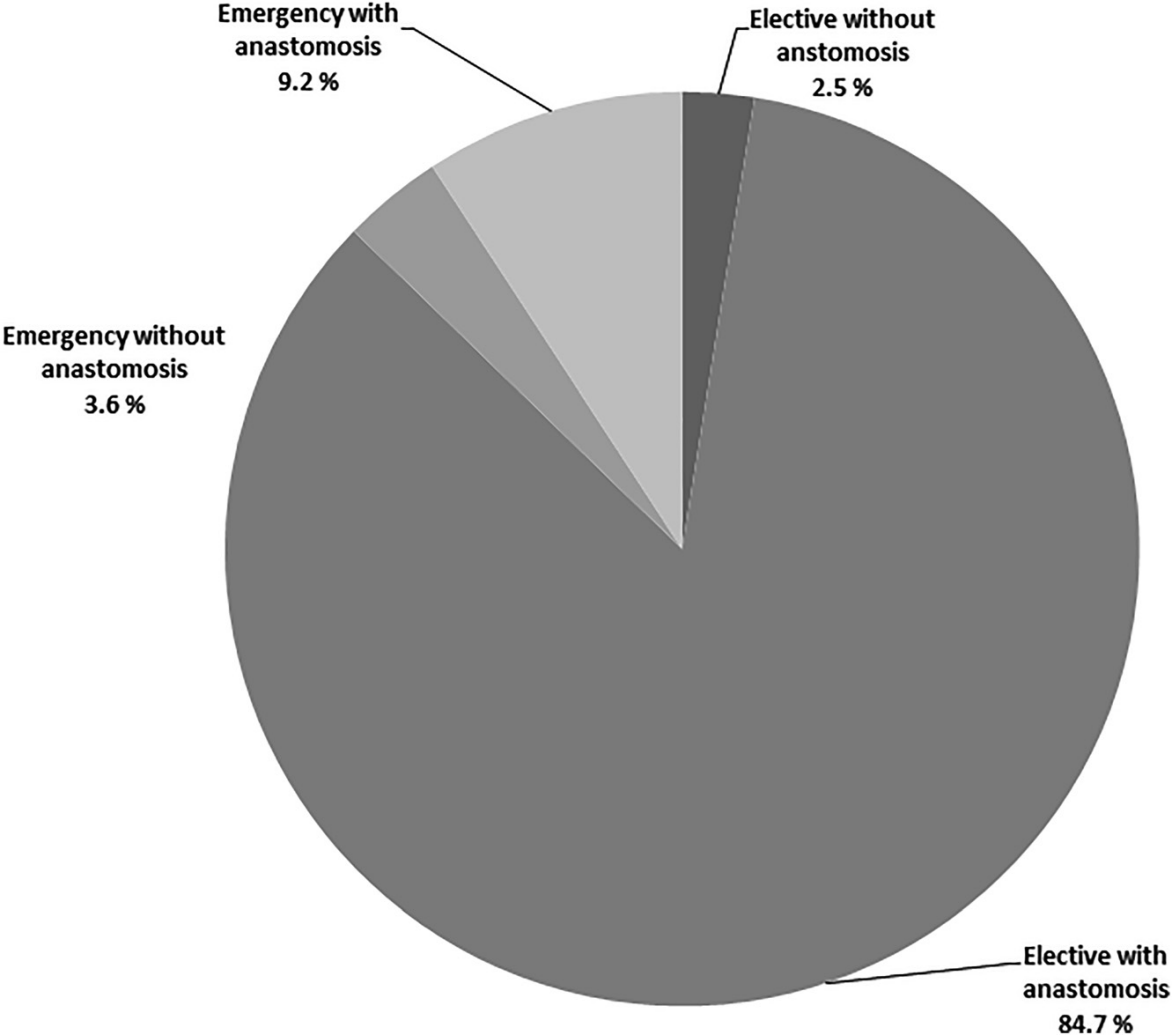
Relative proportions of patients with different operation techniques and urgency.

From 2010 to 2016, data from a total of 14,466 patients were documented (mean age, 72.8 [range, 22–96] years; sex ratio, m:f=7,696:6,770). From this group, 717 patients (4.96 %) were included in a matched-pair analysis. The majority of these patients underwent elective surgery (*n*=12,620 patients; 87.2 %) regardless of whether a bowel anastomosis or an ostomy was created. In emergency surgery, a bowel anastomosis was possible in a large proportion (*n*=1,332 patients [72.1 %]). In contrast, 514 patients (27.9 %) who had undergone emergency surgery received an ostomy. An ostomy had to be created even less frequently in patients who had undergone planned surgery (*n*=366 [2.5 %]; Figure 1).

### Early postoperative outcomes

#### Mortality

Cases of postoperative mortality were mainly due to general complications. Table 1 shows that 5.1 % of all patients died from general, 2.6 % from surgical complications, and 3.0 % from their pre-existing condition (p<0.01). Furthermore, among the patients treated in an emergency without intestinal anastomosis, a high proportion died from their pre-existing condition (17.0 %). Among the patients treated electively, the proportions were 1.6 % (with anastomosis) and 8.2 % (without anastomosis) (p<0.01).

**Table 1:** Cause of death depending on surgical technique and urgency.

Cause of death	Elective without anastomosis		Elective with anastomosis		Emergency without anastomosis		Emergency with anastomosis		In total		p value
	*n*	%	*n*	%	*n*	%	*n*	%	*n*	%	
Surgical complications	1	0.9 %	112	2.3 %	10	4.9 %	29	5.1 %	152	2.6 %	<0.001
General complications	11	8.9 %	202	4.0 %	33	15.1 %	59	10.1 %	305	5.1 %	
Preexisting condition	10	8.2 %	78	1.6 %	38	17.0 %	50	8.6 %	176	3.0 %	

Both patients who underwent ostomy creation or emergency surgery had a worse risk profile (risk factors such as arterial hypertension, diabetes mellitus, and secondary cardiac or renal diseases).

#### General postoperative complications

Table 2 shows that there was a higher incidence of general postoperative complications in emergency surgeries and elective procedures without anastomosis. The most common in this study were cardiac and pulmonary complications and urinary tract infections, among which significant differences were found.

**Table 2:** General postoperative complications depending on surgical technique.

General postoperative complications	Elective without anastomosis		Elective with anastomosis		Emergency without anastomosis		Emergency with anastomosis		p value
	*n*	%	*n*	%	*n*	%	*n*	%	
Minimum one	109	29.5 %	2,072	16.8 %	174	33.7 %	376	28.1 %	<0.001
None	260	70.5 %	10,231	83.2 %	342	66.3 %	962	71.9 %	<0.001
One	76	20.6 %	1,420	11.5 %	107	20.7 %	214	16.0 %	
Two	17	4.6 %	440	3.6 %	26	5.0 %	83	6.2 %	
3 and more	16	4.3 %	212	1.7 %	41	7.9 %	79	5.9 %	
Pulmonal	25	6.8 %	366	3.0 %	47	9.1 %	108	8.1 %	<0.001
Pneumonia	22	6.0 %	453	3.7 %	53	10.3 %	122	9.1 %	<0.001
Urethral infection	13	3.5 %	376	3.1 %	28	5.4 %	43	3.2 %	0.026
Fever	10	2.7 %	267	2.2 %	20	3.9 %	51	3.8 %	<0.001
Cardial	37	10.0 %	463	3.8 %	47	9.1 %	102	7.6 %	<0.001
Multi-organ failure	9	2.4 %	118	1.0 %	38	7.4 %	51	3.8 %	<0.001
Thrombosis	4	1.1 %	30	0.2 %	6	1.2 %	7	0.5 %	<0.001
Renal	14	3.8 %	198	1.6 %	25	4.8 %	56	4.2 %	<0.001
Other	29	7.9 %	703	5.7 %	41	7.9 %	104	7.8 %	0.002

Complications occurred much less frequently in cases of surgery with anastomosis than in cases of ostomy creation. This proportion (16.8 %) was particularly clearly undercut in planned operations. In emergency cases with anastomosis, complications occurred in 28.1 %. Among elective procedures performed without intestinal anastomosis, 29.5 % were affected by postoperative complications. The largest proportion of patients (33.7 %) was affected by complications in emergency cases treated with ostomy creation (p<0.01).

It can be concluded that electively treated patients who had received anastomosis were the least likely to experience a common postoperative complication, while those who had received urgent care without anastomosis had the highest risk of experiencing a general postoperative complication.

#### Specific postoperative complications

Approximately one quarter of patients are likely to have a specific postoperative complication, most frequently wound infection, sepsis, atony, or abdominal pain. If the general and specific postoperative complications are compared, parallels become apparent: Similar to the general postoperative complications, elective procedures with anastomosis placement had the lowest risk of a special postoperative complication. A total of 22.2 % of the electively operated patients who had received an anastomosis experienced a specific postoperative complication. In contrast, if elective surgery was performed without an anastomosis, 26.8 % experienced specific complications (p<0.01). Patients who underwent emergency anastomosis had a 36.2 % risk of complications. Patients who had undergone emergency surgery without anastomosis had a 43.4 % rate of complications. This represented 21.2 % more patients than electively treated patients who received an intestinal anastomosis (22.2 %; p*<*0.01) (Table 3).

**Table 3:** Specific postoperative complications in comparison with urgency and surgical technique.

Specific postoperative complications	Elective without anastomosis		Elective with anastomosis		Emergency without anastomosis		Emergency with anastomosis		In total	
	*n*	%	*n*	%	*n*	%	*n*	%	*n*	%
Minimum one	99	26.8 %	2,734	22.2 %	224	43.4 %	484	36.2 %	3,539	24.4 %
None	270	73.2 %	9,568	77.8 %	292	56.6 %	854	63.8 %	10,984	75.6 %
Bleeding	6	1.6 %	110	0.9 %	9	1.8 %	8	0.6 %	133	0.9 %
Abscess	13	3.5 %	221	1.8 %	27	5.3 %	32	2.4 %	293	2.0 %
Sepsis	7	1.9 %	152	1.2 %	40	7.8 %	42	3.2 %	241	1.7 %
Anastomotic insufficiency	0	0.0 %	673	5.5 %	0	0.0 %	112	8.5 %	785	5.5 %
Aseptic wound healing disorder	14	3.8 %	279	2.3 %	23	4.5 %	52	3.9 %	368	2.5 %
Wound infections	27	7.4 %	551	4.5 %	67	13.1 %	99	7.5 %	744	5.2 %
Intrabdominal abscess	6	1.6 %	109	0.9 %	9	1.8 %	19	1.4 %	143	1.0 %
Mechanic ileus	4	1.1 %	122	1.0 %	6	1.2 %	12	0.9 %	144	1.0 %
Fistula	3	0.8 %	35	0.3 %	2	0.4 %	5	0.4 %	45	0.3 %
Peritonitis	6	1.6 %	216	1.8 %	19	3.7 %	54	4.1 %	295	2.0 %
Atony	21	5.7 %	630	5.2 %	31	6.1 %	117	8.8 %	799	5.5 %
Burst abdomen	16	4.4 %	300	2.5 %	30	5.9 %	64	4.8 %	410	2.8 %
Multi-organ failure	1	0.3 %	66	0.5 %	13	2.5 %	27	2.0 %	107	0.7 %
Stoma complications	7	1.9 %	9	0.1 %	15	2.9 %	3	0.2 %	34	0.2 %
Other	14	3.8 %	443	3.6 %	35	6.8 %	66	5.0 %	558	3.9 %

Furthermore, anastomotic insufficiencies develop more frequently from emergency surgeries than from planned procedures. In this study, 8.5 % of those patients, who had undergone emergency surgery vs. 5.5 % of those who had undergone elective surgery developed anastomotic insufficiency (p<0.01). Consequently, urgency can be identified as a risk factor for the development of a specific postoperative complication. In contrast, for the risk of general postoperative complications, surgical technique was the main risk factor.

In summary, complications occurred less frequently when an anastomosis was performed but this is usually caused by the pre-existing condition and the patient’s risk profile, which influence surgical technique and recovery. Therefore, surgical technique alone cannot be held responsible for the occurrence of complications.

### Long-term oncological outcomes

#### Tumor stage

**Figure 2:**
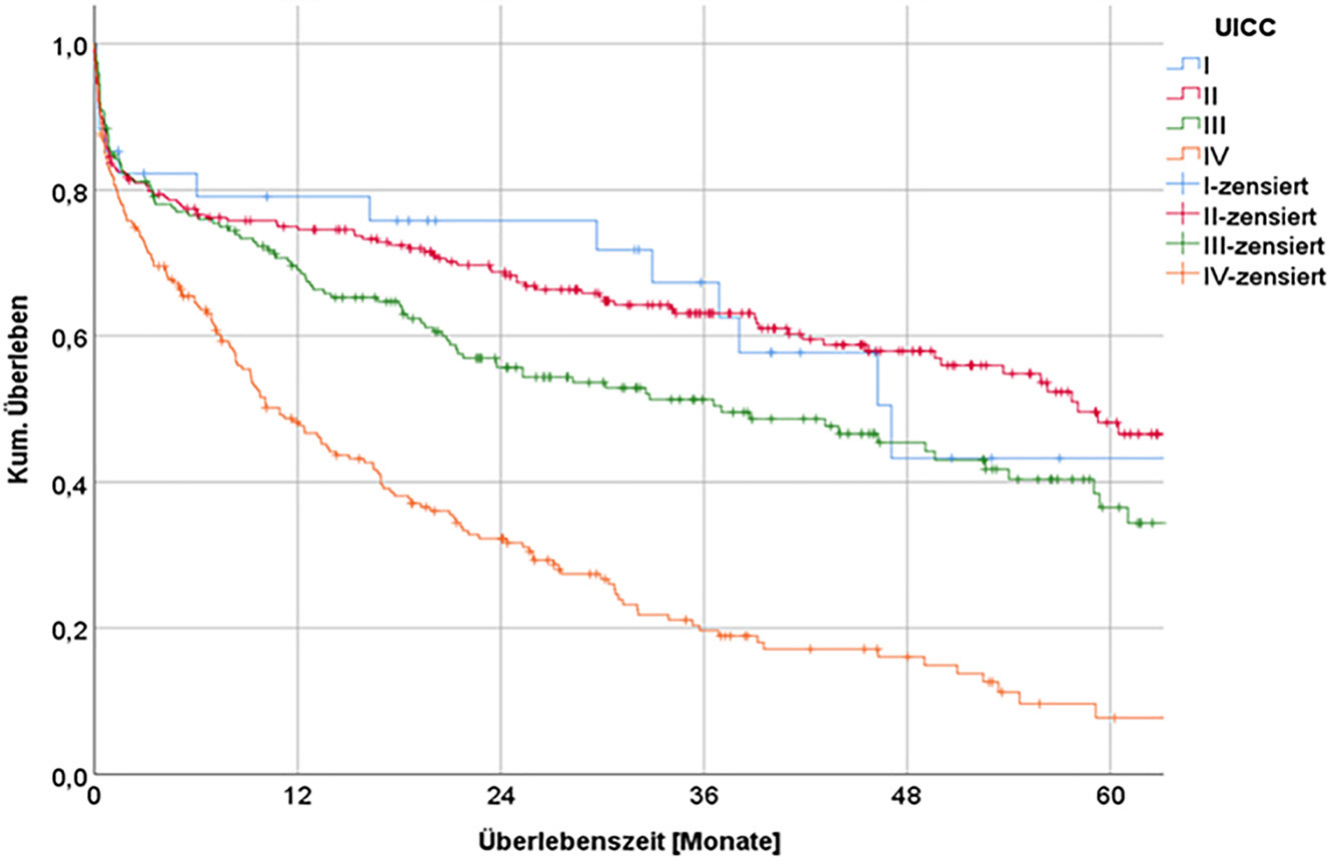
Long-term survival depending on UICC-stage.

The most important factors influencing long-term survival were age, resection status, and tumor stage (according to TNM and UICC). The more advanced the tumor growth according to its classification, the lower the long-term survival (Figure 2). The median survival time of patients with UICC stage IV cancer was 16.4 months. After 60 months, 79.0 % of the UICC-stage I patients were still alive vs. 65.9 % of those with UICC stage II, 57.7 % of those with UICC stage III, and 13.7 % of those with UICC stage IV (Table 4).

**Table 4:** Significant difference – long-term survival of different UICC-stages.

Significant differences – UICC		UICC I	UICC II	UICC III	UICC IV
No anastomosis	UICC I		0.394	0.491	0.057
	UICC II	0.394		0.913	0.000
	UICC III	0.491	0.913		0.000
	UICC IV	0.057	0.000	0.000	
Anastomosis	UICC I		0.576	0.043	0.000
	UICC II	0.576		0.002	0.000
	UICC III	0.043	0.002		0.002
	UICC IV	0.000	0.000	0.002	

#### Surgical technique

**Figure 3:**
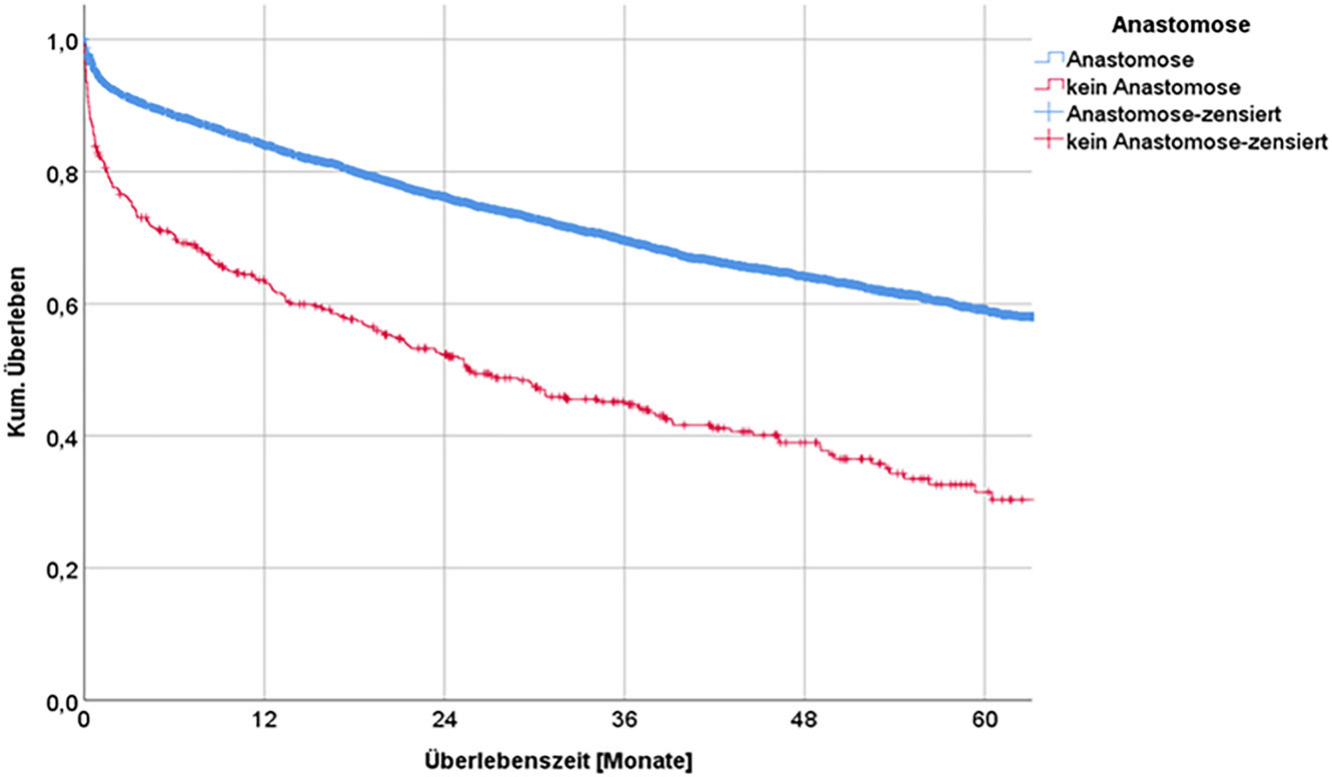
Long-term survival depending on surgical technique.

Comparison of surgical techniques revealed large differences. In patients with ostomy, the median survival time was only 25.7 months. In patients with an anastomosis, the median survival time was longer than the observation period of 60 months. The difference in survival time according to surgical technique was more than 3 years. Regarding the 5-yr-overall survival rate, 59.1 % of the patients with anastomosis were alive after 60 months vs. only 31.5 % without anastomosis, showing a statistically significant difference (Figure 3). In addition, the considerable postoperative mortality in patients with restored intestinal continuity, i.e., without anastomosis, is striking (Table 5).

**Table 5:** Significant difference – long-term survival of different urgencies.

Significant differences – urgency		Elective	Emergency
No anastomosis	Elective		0.954
	Urgency	0.954	
Anastomosis	Elective		0.150
	Urgency	0.150	

#### Operative urgency

Urgency also plays a major role in survival. The median survival time was 29.9 months in patients who had undergone emergency operation or urgent surgery. The 5-yr-survival rate was 60.1 % for elective surgery vs. 37.7 % for urgent surgery.

**Figure 4:**
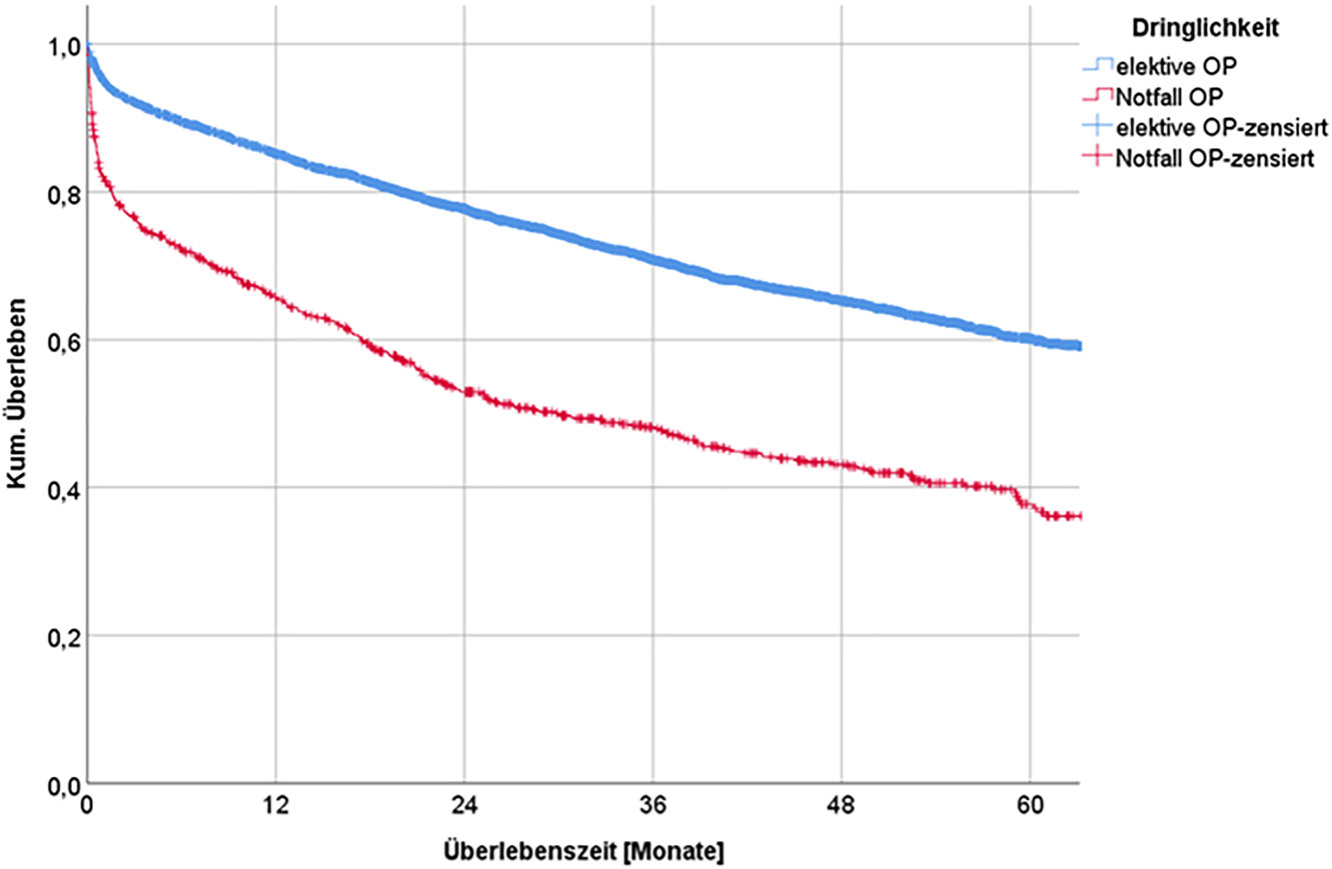
Long-term survival depending on urgency.

Accordingly, patients with the same tumor stage, age, and risk factors had a better chance of survival if they had undergone elective surgery or intestinal anastomosis or were elderly (Table 6, Figure 4).

**Table 6:** Significant difference – long-term survival of different surgical techniques.

Significant differences – surgical technique	Anastomosis	No anastomosis
Anastomosis		0.18
No anastomosis	0.18	

### Factors influencing long-term survival (Table 7)

In summary, the following negative factors influencing survival were identified: older age, several risk factors, advanced tumor growth or higher tumor stage (pT, pN, M1, and not reached R0 status [each p*=*0.000]), no anastomosis (p=0.011), male sex (p=0.001), and emergency surgery (p*=*0.000).

Multivariate analysis revealed that older patients (p*=*0.000) and those with distant metastases benefit more from ostomy than those in the same age group or with a given M1 status, in whom an intestinal anastomosis was attempted (p=0.016) (Table 8A and B). The remaining patients significantly benefited from an anastomosis.

**Table 7:** Influencing factors for long-term survival (95 %-confidence interval).

	p value	Hazard ratio (HR)	95.0 % CI
Age groups			

Age groups 1 vs. 2	0.168	1.124	[0.952; 1.327]
Age groups 1 vs. 3	0.000	1.740	[1.523; 1.987]
Age groups 1 vs. 4	0.000	3.293	[2.862; 3.788]

Risk factors			

0 vs. 1	0.000	1.388	[1.204; 1.6]
0 vs. 2	0.000	1.639	[1.414; 1.9]
0 vs. 3	0.000	2.798	[2.404; 3.256]

pT stage			

pT1 vs. pTis	0.956	0.975	[0.396; 2.403]
pT1 vs. pT2	0.854	1.087	[0.447; 2.647]
pT1 vs. pT3	0.391	1.470	[0.609; 3.548]
pT1 vs. pT4	0.073	2.247	[0.928; 5.445]

pN stage			

pN0 vs. pN1	0.000	1.262	[1.123; 1.418]
pN0 vs. pN2	0.000	1.765	[1.567; 1.989]

M stage			

M0 vs. M1	0.000	2.310	[1.927; 2.769]

R status			

R0 vs. R1	0.000	1.731	[1.363; 2.198]
R0 vs. R2	0.000	1.657	[1.375; 1.996]

Anastomosis			

Anastomosis vs. no anastomosis	0.011	1.225	[1.047; 1.433]

Sex			

Female vs. male	0.001	1.167	[1.069; 1.274]

Urgency			

Elective vs. emergency	0.000	1.617	[1.429; 1.829]

Age group 1–4 and risk factors 0–3 represent semiquantitative classifications from the youngest to the oldest group of patients and no to a substantial number of risk factors, respectively.

**Table 8:** (A and B) Influencing factors for long-term survival of study patients with colon CA resection: (A) with colon anastomosis. (B) Without colon anastomosis.

	p value	Hazard ratio (HR)	95.0 % CI
(A) With colon anastomosis			

Age groups			

Age groups 1 vs. 2	0.294	1.409	[0.743; 2.671]
Age groups 1 vs. 3	0.002	2.244	[1.348; 3.736]
Age groups 1 vs. 4	0.000	4.427	[2.614; 7.497]

Risk factors			

0 vs. 1	0.461	1.230	[0.709; 2.134]
0 vs. 2	0.698	1.116	[0.642; 1.939]
0 vs. 3	0.002	2.417	[1.368; 4.272]

pT stage			

pT1 vs. pT2	0.594	1.774	[0.215; 14.617]
pT1 vs. pT3	0.839	1.229	[0.167; 9.064]
pT1 vs. pT4	0.473	2.081	[0.281; 15.394]

pN stage			

pN0 vs. pN1	0.561	1.132	[0.746; 1.718]
pN0 vs. pN2	0.006	1.762	[1.174; 2.644]

M stage			

M0 vs. M1	0.016	2.030	[1.14; 3.615]

R status			

R0 vs. R1	0.071	1.981	[0.943; 4.165]
R0 vs. R2	0.301	1.346	[0.766; 2.363]

Urgency			

Elective vs. emergency	0.003	1.618	[1.175; 2.228]

(B) Without colon anastomosis			

Age groups			

Age groups 1 vs. 2	0.341	0.764	[0.439; 1.329]
Age groups 1 vs. 3	0.036	1.594	[1.03; 2.468]
Age groups 1 vs. 4	0.000	3.277	[2.108; 5.094]

Risk factors			

0 vs. 1	0.268	1.392	[0.775; 2.499]
0 vs. 2	0.166	1.536	[0.836; 2.822]
0 vs. 3	0.002	2.692	[1.451; 4.996]

pT stage			

pT1 vs. pT2	0.114	0.276	[0.056; 1.364]
pT1 vs. pT3	0.019	0.166	[0.037; 0.743]
pT1 vs. pT4	0.090	0.273	[0.061; 1.223]

pN stage			

pN0 vs. pN1	0.748	0.942	[0.653; 1.358]
pN0 vs. pN2	0.007	1.678	[1.151; 2.448]

M stage			

M0 vs. M1	0.046	1.680	[1.009; 2.796]

R status			

R0 vs. R1	0.023	2.081	[1.107; 3.914]
R0 vs. R2	0.029	1.797	[1.063; 3.039]

Urgency			

Elective vs. emergency	0.027	1.385	[1.037; 1.849]

Age group 1–4 and risk factors 0–3 represent semiquantitative classifications from the youngest to the oldest group of patients and no to a substantial number of risk factors, respectively.

## Discussion

Major factors influencing long-term survival are differentiation and tumor stage (G, pT, pN, M, R status, UICC stage), patient age, number of risk factors, and complication rate.

An important finding of this work is that – in particular – postoperative 30-d mortality is an important factor influencing long-term survival. This is often due to peri- and post-operative complications. On the one hand, such complications are associated with increased risk factors; on the other hand, they occur more frequently in cases of discontinuity resection and emergency surgery.

### Operative aspects and methodological study considerations

To consider these influencing factors and results independently, the formation of matched pairs and the multivariate analysis were crucial. Matched-pairs analysis with respect to pT, pN, R, and M status; UICC stage; grading; risk factors; age; sex; BMI; and colon CA location (forming a “statistical twin group”) allowed the comparison of patients surgically resected for colon CA:

(i) with vs. without intestinal anastomosis (as well as)
(ii) on an elective vs. emergency basis

for determination of the effects of surgical technique and urgency on early postoperative (rather surgical) results and long-term (rather oncological) prognosis.

### Early postoperative outcome

The association of intraoperative and postoperative complications with increased postoperative mortality is in line with findings of current studies. In a multicenter observational study, Marusch et al. [10] found that patients who died postoperatively suffered significantly more frequently from general and specific postoperative complications. The same conclusion was also reached in the prospective study of Clauer et al. The major factors influencing overall survival included age, UICC stage, R status, lymph node involvement, cardiovascular disease, and the occurrence of surgery-specific complications [11]. The association of general and surgery-specific complications with certain pre-existing conditions has been demonstrated by various authors. For example, Legler [12] confirmed the association between preoperative concomitant diseases and general as well as surgical complications. In addition, almost all patients who died postoperatively had a concomitant disease. These results are in line with those of current studies. Kirchhoff et al. [9] and Alves et al. [13] also reported an association between postoperative complications and worse pre-existing condition (as measured by “American Society of Anesthesiologists” score). Advanced age also appears to be a significant factor influencing the occurrence of complications [9, 13, 14]. The present study demonstrated the associations between preoperative risk factors and postoperative complications and between complication rate and mortality.

Another important factor influencing the development of complications is the intervention’s urgency. Various studies have shown that emergency interventions (due to ileus, intestinal perforation, etc.) are associated with an increased risk of postoperative complications [15, 16]. The anastomotic insufficiency rate in Legler’s study was 5.4 % for elective surgery vs. 21 % for emergency surgery [12]. In this regard, the current study’s findings support those in the literature. Due to the high complication rate in urgent colon resections, ostomy creation may be considered in these situations. Meyer et al. [17] concluded in a prospective study that this was a good way to perform an emergency colon resection. In particular, this appeared to be a favorable option in cases of ileus or bowel perforation, as it was associated with lower mortality than other radical colon resections. Here, the study results differ. Although emergency procedures are generally associated with more complications than elective procedures, patients in emergency settings also appear to benefit from anastomosis creation vs. discontinuity maintenance (no anastomosis creation), which is longer and more prone to complications. In addition, ostomy creation is advantageous in palliative situations since the postoperative mortality rate is lower in such cases [18].

An interesting further question would be, on the one hand, which complications lead to death and how often. On the other hand, the causes of death of patients without postoperative complications could be analyzed in more detail.

### Long-term oncological outcome

The Kaplan–Meier curves for surgical technique and urgency showed significant differences in survival time in the matched-pair analysis, although not with major differences. One reason for this could be the matched-pair analysis itself: Since the “twin groups” differed in only one characteristic (anastomosis vs. none or emergency vs. elective), many patients with a very good perioperative condition and few or no risk factors were excluded because they are rarely treated with ostomy creation. It stands to reason that postoperative mortality rates are high in both types of surgery, as these are patients with poorer preoperative general conditions. Nevertheless, the differences in surgical techniques and urgency are statistically significant (p*=*0.00). Accordingly, patients with the same tumor stage, age, and risk factors have a better chance of survival if they are operated on electively or with intestinal anastomosis based on the risk profile assessed as acceptable; in other words, the creation of an intestinal anastomosis and elective surgery are prognostically favorable factors in colon CA resection. The multivariable analysis confirmed this with the exception of older patients and those with distant metastases.

This finding is in line with those of De Simone et al. [19]. In a retrospective study, the authors found that patients with a more disadvantageous general condition (measured semiquantitatively by “American Society of Anesthesiologists” score) and acute symptoms benefit from a shorter operating time due to ostomy creation and avoidance of the more complication-prone anastomosis placement. Ansaloni et al. recommended that patients at high surgical risk should be treated with ostomy placement to avoid anastomotic insufficiency [20] or prevent it completely. Wong et al. confirmed that ostomy creation is performed more frequently in the elderly [21], but the influence of distant metastasis could not be confirmed in the current literature. However, the aforementioned studies did not provide specific details on a rather reduced general condition and high risk of surgery. The exact role of distant metastasis in this context cannot be completely determined, although it is precisely this specific aspect that seems to be of extraordinary interest in the current work; in general, the presented data otherwise concur with those of the current literature that, under adequate risk assessment and exhaustive surgical preparation (prehabilitation), intestinal anastomosis should generally be the aim to effectively pursue long-term survival and ensure the best possible prognosis. An exception is made for elderly patients or those with distant metastases who, due to an increased intraoperative and postoperative complication potential (secondary disease profile, concomitant medication, and limited organ reserve; catabolic metabolic state, and immunosuppressive effects in advanced tumor stage as in metastatic tumor growth), require special attention with respect to:

– longer lasting surgical interventions for restoring intestinal continuity after resection by anastomosis;
– in case of urgent or emergency surgery (limited surgical preparation, increased risk of bleeding); and
– compensation requirement for blood products, high proportions of a given pre-medication (inhibition of platelet aggregation, anticoagulation, etc.).

The therapeutic recommendation for patients with distant metastases has not yet been described in the literature, and it is relevant in the surgeon’s decision-making process.

### Strengths

– Representative sample
– Prospective clinical systematic multicenter observational study design
– Matched-pair analysis
– Displayed surgical quality assurance for the reflection of the abdominal-surgery everyday life in clinical practice and as a contribution to research on clinical care

### Limitations

The study has several limitations. First, it is based on registry data, which are subject to bias that may distort the interpretation of our results. To finally clarify the questions discussed above, randomized controlled studies are required. Although the formation of matched pairs is intended to allow better comparability, this could become a problem. Since the “twin groups” differ only in one characteristic (anastomosis vs. none or emergency vs. elective), many patients with a very good perioperative condition and few risk factors were excluded because they rarely underwent anus-praeter creation. Thus, it stands to reason that postoperative lethality rates are high in both surgical methods, as these patients have a poorer preoperative general condition.

## Conclusions

Based on the current study results, the elective resection of a tumor with intestinal anastomosis placement is recommended for patients with histologically confirmed colon CA after an adequate risk assessment and exhaustive surgical preparation (prehabilitation) since it is associated with the best long-term survival and can, thus, be considered a prognostically favorable factor for long-term survival. In particular, the decision to proceed in an emergency setting should be subordinate to this, e.g., by overcoming an emergency ileus using passive ostomy placement and appropriate prehabilitation measures (high-caloric nutrition, cardiopulmonary conditioning, physiotherapy, breathing exercises etc.) and subsequent elective colon-CA resection after stabilization for pre-surgical condition to achieve the best possible long-term outcomes.

In case of distant metastatic tumor growth of colon CA or patients with colon CA of advanced age, ostomy creation should be carefully considered due to the more severe influence of:

– distant metastasis and age-associated secondary diseases;
– the more critical initial state; and
– the higher risk of complications at a more advanced age.

### Summary (learning points)

– In principle, the elective creation of an anastomosis is recommended in histologically confirmed colon CA.
– In colon CA patients of advanced age or with distant metastases, critical consideration of treatment options is required, in particular, with regard to the creation of an ostomy.
– The most important prognostic factor for long-term survival is early postoperative mortality.
– Early postoperative mortality is characterized by postoperative complications, most of which can be attributed to preoperative condition and risk factors.
